# Balloon Enteroscopy-Assisted Endoscopic Retrograde Cholangiopancreatography for the Treatment of Common Bile Duct Stones in Patients with Roux-en-Y Gastrectomy: Outcomes and Factors Affecting Complete Stone Extraction

**DOI:** 10.3390/jcm10153314

**Published:** 2021-07-27

**Authors:** Taisuke Obata, Koichiro Tsutsumi, Hironari Kato, Toru Ueki, Kazuya Miyamoto, Tatsuhiro Yamazaki, Akihiro Matsumi, Yuki Fujii, Kazuyuki Matsumoto, Shigeru Horiguchi, Kengo Yasugi, Tsuneyoshi Ogawa, Ryuta Takenaka, Hiroyuki Okada

**Affiliations:** 1 Department of Gastroenterology, Okayama University Hospital, Okayama 7008558, Japan; p47691mh@s.okayama-u.ac.jp (T.O.); katou-h@cc.okayama-u.ac.jp (H.K.); ty1114db@gmail.com (T.Y.); akihiro.matsumi.gastro@gmail.com (A.M.); y_f1105@yahoo.co.jp (Y.F.); matsumotokazuyuki0227@yahoo.co.jp (K.M.); horiguchis@gmail.com (S.H.); hiro@md.okayama-u.ac.jp (H.O.); 2 Department of Internal Medicine, Fukuyama City Hospital, Fukuyama 7218511, Japan; ueki0041@fchp.jp (T.U.); ppur0jyn@s.okayama-u.ac.jp (K.Y.); t-ogawa@xa3.so-net.ne.jp (T.O.); 3 Department of Internal Medicine, Tsuyama Chuo Hospital, Okayama 7080841, Japan; ttpcx442@yahoo.co.jp (K.M.); rtakenak@gmail.com (R.T.)

**Keywords:** bile duct stone, endoscopic retrograde cholangiography, Roux-en-Y anastomosis, short-type balloon enteroscopy, complete stone removal, gastrectomy

## Abstract

Background: Endoscopic retrograde cholangiopancreatography (ERCP) for extraction of common bile duct (CBD) stones in patients with Roux-en-Y gastrectomy (RYG) remains technically challenging. Methods: Seventy-nine RYG patients (median 79 years old) underwent short-type double-balloon enteroscopy-assisted ERCP (sDBE-ERCP) for CBD stones at three referral hospitals from 2011–2020. We retrospectively investigated the treatment outcomes and potential factors affecting complete stone extraction. Results: The initial success rates of reaching the papilla of Vater, biliary cannulation, and biliary intervention, including complete stone extraction or biliary stent placement, were 92%, 81%, and 78%, respectively. Of 57 patients with attempted stone extraction, complete stone extraction was successful in 74% for the first session and ultimately in 88%. The adverse events rate was 5%. The multivariate analysis indicated that the largest CBD diameter ≥ 14 mm (odds ratio (OR), 0.04; 95% confidence interval (CI), 0.01–0.58; *p* = 0.018) and retroflex position (OR, 6.43; 95% CI, 1.12–36.81; *p* = 0.037) were independent predictive factors affecting complete stone extraction achievement. Conclusions: Therapeutic sDBE-ERCP for CBD stones in a relatively elderly RYG cohort, was effective and safe. A larger CBD diameter negatively affected complete stone extraction, but using the retroflex position may be useful for achieving complete stone clearance.

## 1. Introduction

Cholelithiasis is an adverse event in patients with surgically altered anatomies due to a history of gastrectomies, such as Billroth-II reconstruction and Roux-en-Y (R-Y) anastomosis [1,2,3]. Since common bile duct (CBD) stones often cause patients life-threatening severe cholangitis and pancreatitis, biliary intervention, such as stone extraction or biliary drainage, is required [4,5]. However, endoscopic treatment of CBD stones via the papilla of Vater is technically challenging, especially in patients who have undergone R-Y gastrectomy (RYG), due to the difficulty of not only reaching the papilla but also performing biliary cannulation or ampullary procedures or stone extraction [6,7,8,9,10], compared to those with normal anatomy. Thus, percutaneous transhepatic intervention or surgery is often performed as an alternative treatment [11,12,13,14].

Recently, balloon enteroscopy-assisted endoscopic retrograde cholangiopancreatography (BE-ERCP) has been reported to be a useful method for post-operative biliary or pancreatic diseases in patients with such surgically altered anatomies [15,16,17,18,19,20,21,22,23]. Owing to the improvement of reachability up to the papilla, the extraction of CBD stones as well as biliary stent placement have been facilitated using this innovative enteroscopy procedure. However, little is known about the detailed outcomes of this treatment in RYG patients, and the factors affecting CBD stone clearance have not been investigated.

In the present study, we clarified the efficacy and safety of BE-ERCP for the treatment of CBD stones in patients with RYG and identified the predictive factors for complete stone extraction.

## 2. Materials and Methods

### 2.1. Study Design

This was a multi-center retrospective study conducted in three tertiary hospitals. This study was approved by the ethics committee at each institution.

### 2.2. Patients

Among the total of 699 patients (1846 sessions) who underwent BE-ERCP between January 2010 and December 2020 at Okayama University Hospital, Fukuyama City Hospital or Tsuyama Chuo Hospital, 79 (11%) who had previously undergone total gastrectomy or subtotal gastrectomy with R-Y anastomosis and had received initial BE-ERCP for the treatment of CBD stones were included in this study. Before BE-ERCP, all patients received blood tests and underwent imaging examinations, such as abdominal ultrasonography, computed tomography, or magnetic resonance cholangiopancreatography, to investigate the suspected CBD stones. Written informed consent was obtained from all patients.

### 2.3. BE-ERCP Procedure

All BE-ERCP procedures were performed using a short-type double-balloon enteroscope (DBE; EI-530B or EI-580BT; Fujifilm, Tokyo, Japan) with a 2.8- or 3.2-mm working channel and a 152-cm working length and a transparent cap attached to its tip, by skilled endoscopists with extensive experience in performing ERCP for patients with normal anatomy. All patients were admitted to each hospital and were in the prone position under conscious sedation with propofol, midazolam, diazepam, or pethidine hydrochloride during the procedure. In addition, all of these procedures were performed under CO_2_ insufflation.

The scope was perorally advanced toward the papilla of Vatar beyond the R-Y anastomosis [24]. After reaching the papilla, biliary cannulation and cholangiography were generally attempted using a catheter (PR-V220Q; Olympus Medical Systems, Tokyo, Japan or MTW ERCP catheter; Medizin-Technische-Werkstätte, Wesel, Germany) with a 0.025-inch guidewire (VisiGlide2; Olympus Medical Systems or RevoWave; Piolax Medical Devices, Kanagawa, Japan). Following confirmation of filling defect suspected of being CBD stones, endoscopic sphincterotomy (EST), precutting, endoscopic papillary balloon dilation (EPBD), and/or endoscopic papillary large balloon dilation (EPLBD; ≥12 mm) [4,16,17,25,26,27,28,29] was performed using a sphincterotome (RotacutII; Medi-Globe GmbH, Achenmühle, Germany or TRUEtome; Boston Scientific, MA, USA), a needle-knife (KD-10Q-1; Olympus Medical Systems) and/or a balloon dilation catheter (ZARA; Century Medical Inc., Tokyo, Japan or GIGA2; Century Medical Inc., Tokyo, Japan). For stone extraction, a retrieval balloon catheter (Tri-Ex; Cook Medical, Tokyo, Japan), basket catheter (Flower Basket V 8-wire type; Olympus Medical Systems), and mechanical lithotripter (ML) (Crusher Catheter; Xemex, Tokyo, Japan or LithoCrushV BML-V437QR-30; Olympus Medical Systems) were usually used. Prophylactic administration of ulinastatin was performed for the prevention of post-ERCP pancreatitis in all patients.

In patients in whom scope insertion to the papilla or biliary cannulation failed, the second BE-ERCP or alternative approach, including surgery, endoscopic ultrasound-guided biliary drainage (EUS-BD) [5,30,31,32,33], percutaneous transhepatic biliary drainage (PTBD), and conservative therapy, was carried out. In some patients with a serious condition or incomplete extraction of CBD stones, endoscopic biliary stenting (EBS) using a 5- to 7-Fr plastic stent was performed for treatment of cholangitis in an initial session; thereafter, complete stone extraction was attempted on readmission.

### 2.4. Definitions

**Figure 1:**
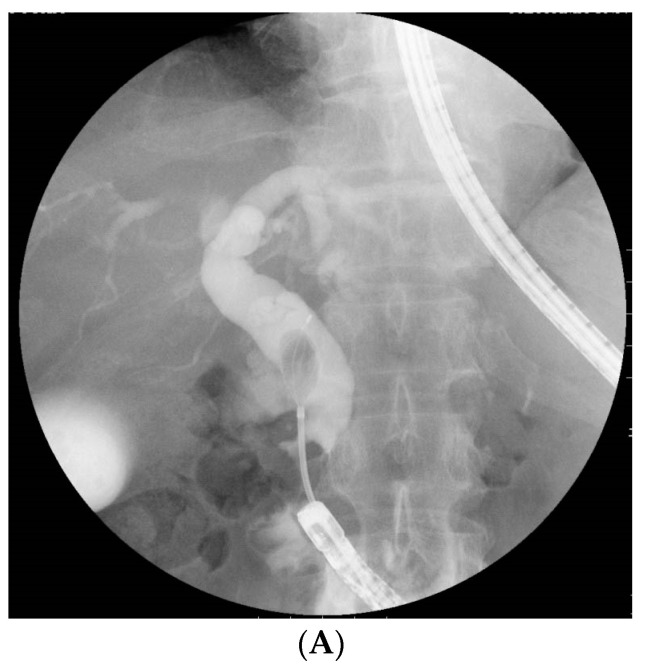

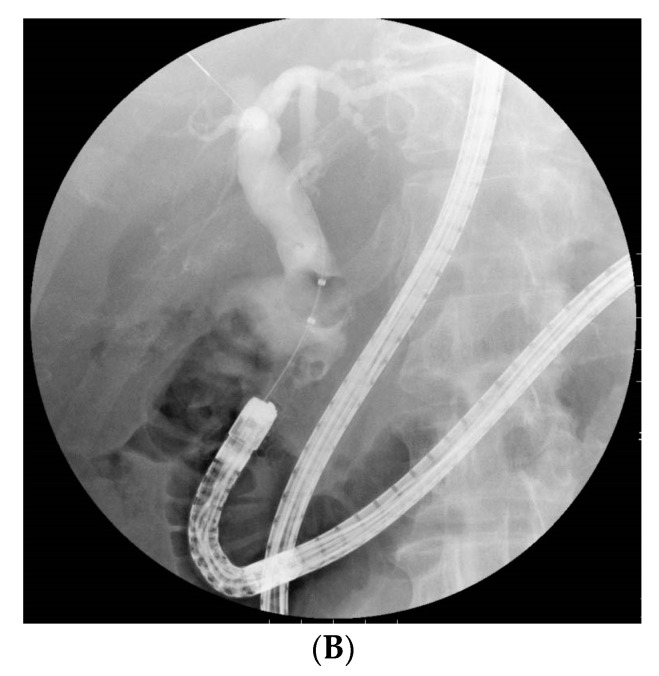
A useful “retroflex position” for stone extraction in a Roux-en-Y gastrectomy patient. (**A**) At the initial session, the scope was stretched after reaching the papilla (not formed the retroflex position). Following successful biliary cannulation, precutting, and endoscopic papillary large balloon dilation, a stone was able to be grabbed with a mechanical lithotripter. However, complete stone extraction was not able to be conducted using any devices, including a basket catheter or balloon catheter, as the axis of the devices did not align with the distal bile duct during the extraction. (**B**) In the second session, the retroflex position was obtained by forming a looped-scope shape. In this manner, the coaxial relationship between the devices and the distal bile duct and a proper distance from the tip of the scope to the papilla of Vater with a better view of the papilla could thus be successfully obtained. This situation allowed stones to be easily removed along the axis of the distal bile duct.

The primary outcome of this study was to reveal the factors affecting complete stone extraction using variables associated with both patient characteristics and procedural contents. The patient-related factors were age, sexuality, the American Society of Anesthesiologists physical status (ASA-PS) classification, diameter of the largest CBD, size of the largest CBD stone, number of stones, and time from RYG to BE-ERCP. Furthermore, the procedure-related factors were initial BE-ERCP, EST/precutting or EPBD/EPLBD, and retroflex position, which was able to provide a better view of the papilla with a J-turn form of the scope at the inferior duodenal angle (IDA) [20] (Figure 1). The secondary outcomes were the technical success rates of initial BE-ERCP, including the rate of reaching the papilla, rate of biliary cannulation, and rate of biliary intervention, such as complete stone extraction and biliary stent placement, as well as adverse events. Complete stone extraction was defined as no detection of residual stones by a cholangiogram. The time to reach the papilla was the duration from the scope insertion to when the papilla was reached. The time to biliary cannulation was the duration from when the papilla was reached to the achievement of biliary cannulation. The total procedural time was defined as the time from scope insertion until withdrawal. Adverse events were defined according to the ASGE guidelines [34].

### 2.5. Statistical Analyses

Continuous variables were expressed as the median and interquartile range (IQR). To identify predictive factors for complete stone extraction, continuous variables were categorized into two groups by the median value, and several factors described above were analyzed in a univariate and multivariate Cox proportional hazard model, along with the odds ratio (OR) and confidence intervals (CIs). The multivariate model included variables with a *p*-value of <0.10 in the univariate model. Statistical significance was considered to be indicated by a *p*-value of <0.05. All analyses were carried out using the JMP (version 15.1.0, SAS Institute Inc., Cary, NC, USA) software program.

## 3. Results

### 3.1. Patients’ Characteristics

All enrolled 79 patients had undergone BE-ERCP for the treatment of CBD stones over a total of 90 sessions. The patient characteristics are shown in Table 1.

The median age was 79 years old, which was considered relatively elderly, and 78% of patients were male. The most common reason for gastrectomy was gastric cancer (91%). Regarding the ASA-PS classification, 57 patients (72%) were classified as ASA-PS 2, while the remaining 22 were ASA-PS 3 or 4. The median diameter of the largest CBD was 14 mm, the median size of the largest CBD stone was 10 mm, and the median number of stones was 2.

### 3.2. Scope Insertion and Biliary Cannulation in an Initial BE-ERCP

Outcomes of initial BE-ERCP for treatment of CBD stones are shown in Table 2. Of the 79 patients, successful scope insertion to the papilla of Vater was obtained in 73 (92%). The reason of unsuccessful scope insertion was the bowel adhesion or long length of R-Y limb, and it took median 64 (IQR, 46–80) mins to discontinue. Subsequent selective biliary cannulation was successfully performed in 64 patients (81%). Of the 15 patients in whom these biliary approaches had failed, surgery was performed in 3 patients, PTBD in 3 patients, and EUS-guided antegrade therapy for stone extraction in 2 patients, while 6 patients were treated with conservative therapy. The remaining patient who had failed biliary cannulation achieved successful cannulation in the second session.

### 3.3. Ampullary Procedure for Stone Extraction at Initial BE-ERCP

Among the 63 patients who achieved a successful cholangiogram, excluding 1 patient in whom the stone had spontaneously passed through the papilla, ampullary procedures were performed for biliary interventions, as shown in Table 3. For stone extraction, EPBD or EPLBD was conducted in 87% (48/55), while EST or precutting alone was performed in 13% (7/55). Of the remaining 8 patients who underwent EBS without stone extraction, 3 (38%) underwent precutting alone, and 5 underwent no ampullary procedure.

### 3.4. Biliary Intervention and Complete CBD Stone Extraction in an Initial BE-ERCP

Of the 63 patients, 42 (53%) received complete stone extraction in a single session. Of the remaining 21 patients, 20 had EBS for drainage due to incomplete CBD stone extraction (*n* = 12), poor maneuverability (*n* = 4), or a poor patient condition (*n* = 4). Another patient failed biliary intervention due to edema of the papilla of Vater and was treated conservatively. Thus, the overall success rate of biliary intervention was 78% (62/79) at the initial BE-ERCP procedure.

### 3.5. Potential Factors Affecting Complete CBD Stone Extraction

CBD stone extraction was ultimately attempted in 66 sessions for 57 patients, including 9 who underwent BE-ERCP twice, due to incomplete extraction at the initial session in 7 and recurrent CBD stone in 2. As a result, complete stone extraction was achieved in 52 sessions (79%). Among the 11 variables examined, the largest CBD diameter ≥ 14 mm (*p* = 0.002) and the largest CBD stone size ≥ 10 mm (*p* = 0.031) were associated with complete stone extraction according to the univariate analysis. In the multivariate analysis, the largest CBD diameter ≥ 14 mm (OR 0.04; 95% CI 0.01–0.58; *p* = 0.018) and retroflex position (OR 6.43; 95% CI 1.12–36.81; *p*  =  0.037) were identified as independent relevant factors for complete stone extraction (Table 4).

Furthermore, among 36 patients with a large CBD diameter (≥14 mm), the retroflex position (*p* = 0.035) was the only potential factor affecting complete stone extraction in univariate analysis (Table 5). Among 39 patients with a large CBD stone size (≥10 mm), the largest CBD diameter ≥ 14 mm (*p* = 0.017) and retroflex position (*p* = 0.037) were significant factors associated with complete stone extraction (Table 6).

### 3.6. Adverse Events

Adverse events were observed in 4 patients (5%; 4/79), including bowel perforation in 2, pancreatitis in 1, and hypoxia in 1 (Table 2). In a patient whose perforation was detected at the IDA after complete stone extraction, a naso-drainage tube was placed around the area, but a high fever with retroperitoneal free air was observed two days later, so laparotomy drainage was performed. The condition gradually improved, but it took 28 days after BE-ERCP before the patient could leave the hospital. The other patient who had small intestinal perforation during scope insertion was able to be treated with double naso-drainage tubes. The mild pancreatitis improved conservatively, with dietary intake delayed one day. Hypoxia occurred in an 83-year-old patient with sepsis (ASA-PS 3) but improved immediately by oxygenation and scope withdrawal after EBS. There were no procedure-related mortalities.

## 4. Discussion

In this study, we retrospectively analyzed the outcomes of therapeutic BE-ERCP for CBD stones in RYG patients treated at three tertiary institutions. Initial biliary intervention, including complete stone extraction or biliary stent placement, was successful in 78% (62/79), complete stone extraction was initially achieved in 53% (42/79) and ultimately in 63% (50/79), and adverse events occurred in 5% (5/79). In addition, we identified two independent factors affecting complete stone extraction: the largest CBD diameter ≥ 14 mm was a negative factor, and the retroflex position was a positive factor, especially in difficult cases with a large CBD diameter or stone size. Thus, this study was the first to clarify the efficacy and safety of therapeutic BE-ERCP for CBD stones in RYG patients and identify the factors affecting complete stone clearance.

To achieve successful endoscopic extraction of CBD stones in patients who had had surgically altered anatomies due to having undergone gastrectomy, such as Billroth-II reconstruction or R-Y anastomosis, four processes needed to be carried out: reaching the papilla of Vater endoscopically, performing selective biliary cannulation, conducting an ampullary procedure (e.g., sphincterotomy or balloon dilation) and performing stone extraction. The first step was considered the most challenging in ERCP for RYG patients due to the excessive length or rigid adhesion of the R-Y limb, especially when using a conventional side-viewing duodenoscope [6] or a forward-viewing colonoscope [7,8,9]. Indeed, successfully reaching the papilla has been reported in 92% (54/59) of Billroth-II patients but only 33–67% of RYG patients. However, due to recent advances in enteroscopes, such as the advent of single-balloon enteroscopy (SBE) as well as DBE, the successful approach rate has remarkably improved to 91–96% in RYG patients with short-type SBE [18,20,22] and 95–98% with short-type DBE [15,19,21]. Similarly, successful scope insertion to the papilla of Vater was obtained in 92% (73/79) of RYG patients using short-type DBE in this study.

Selective biliary cannulation was also challenging due to the difficulty of positioning the scope from a front view of the papilla of Vater and the limited controllability of the catheter through elevator-unequipped enteroscopes, in contrast to standard duodenoscopes. Previous studies reported the success rate of biliary cannulation in RYG patients to be 74–95% [15,18,19,20,21,22]. One of the tips for biliary cannulation is to perform the procedure using the retroflex position, which can facilitate direct visualization of the papilla from the front [20]. Recently, the position was reported to be a potential favorable factor for successful biliary cannulation [22]. In the present study, 12% (9/73) of patients had failed biliary cannulation, but alternative approaches, such as PTBD which might induce pain and discomfort associated with the external transhepatic catheter [11,12,13,14], EUS-BD (including EUS-guided antegrade intervention) which required complicated process for stone extraction and had a risk of biliary peritonitis [5,30,31,32,33], surgery or conservative therapy, improved the situation. Depending on the patient condition and capabilities of the institution, an immediate decision to alter the treatment plan may also be crucial.

The basic strategy for an ampullary procedure and subsequent stone extraction is considered to be the same as for managing patients with normal anatomy. In the present study, several combinations of an ampullary procedure were performed, as shown in Table 3. Given the difficulty of sphincterotomy and the precutting method due to the inverted view, EPBD or EPLBD alone may be acceptable for RYG patients [35], as it is for normal anatomies [36,37] and Billroth-II gastrectomy patients [27]. Regarding stone extraction, the latest enteroscope with a 3.2 mm working channel can utilize most devices, including an ML, and complete stone extraction was ultimately achieved in 88% of patients in the present study in whom such a procedure was attempted. For difficult cases, stone extraction in two sessions following drainage was recommended [4,5]. In addition, in some cases with large CBD stones, electrohydraulic lithotripsy (EHL) using cholangioscopy [4,38,39,40], percutaneous transhepatic cholangioscopy (PTCS) [14], or EUS-guided antegrade cholangioscopy [31,32,33] might need to be considered for complete extraction.

This was the first study to reveal the factors affecting complete stone extraction in RYG patients who underwent BE-ERCP. First, we identified an interesting risk factor for incomplete stone extraction: the largest CBD diameter ≥ 14 mm. Dilation of the CBD in RYG patients is often seen post-cholecystectomy [41]. In patients with a large CBD stone size and large CBD diameter, sufficient papillary dilation by EPLBD with or without crushing stones using an ML was usually required for successful stone extraction [4,16,17,25,26,27,28,29,36,37,42], but achieving stone clearance is not easy. A previous study reported that a large stone size was a risk factor for incomplete stone extraction by ERCP, in patients with a history of Billroth-II [42] as well as those with normal anatomy [43]. In contrast, small stones floating into larger diameter CBD are often difficult to grasp, even when using available devices, such as a basket or retrieval balloon catheter. This was also an issue when large stones were crushed with an ML. Thus, regardless of the CBD stone size, a large CBD diameter can make complete stone extraction difficult. Dedicated devices that can easily catch small stones floating in large diameter CBDs are desired.

In addition, a retroflex position was identified as a positive factor affecting complete stone extraction. As mentioned above, this position was reported to be useful for successful biliary cannulation in RYG patients [20,22]. The retroflex position can be obtained by advancing the endoscope without releasing the looped scope and forming a J-turn at the IDA. Thereby, a coaxial relationship between the devices and distal CBD and maintaining a proper distance from the tip of the scope to the papilla of Vater with a better view of the papilla can thus be obtained. Such a situation can facilitate stones to be removed along the axis of the CBD. In the present study, in a sub-analysis of the difficult-to-manage cohorts—i.e., those with a large CBD diameter (≥14 mm) or stone size (≥10 mm)—the retroflex position was also a significant factor affecting successful complete stone extraction. In fact, we experienced several cases where stone extraction could not be completed initially in the non-retroflex position, whereas the retroflex position allowed complete stone extraction to be easily performed in the second session, as shown in Figure 1. Taken together, these findings suggest that the retroflex position may be recommended for complete stone extraction as well as successful biliary cannulation in RYG patients. However, this technique should be performed carefully due to the risk of perforation at the IDA.

Adverse events occur in 5–18% of patients treated with this procedure [15,20,22,44], and the incidence rate was 5% in the present study. In contrast to conventional ERCP, perforation is one of the most common adverse events for this procedure [45] and occurs mainly during scope insertion or stone extraction. Immediately noticing the issue and thoroughly performing intraluminal drainage is important, as the situation can sometimes be managed if minor perforation occurs, as shown in one of our cases. Acute pancreatitis occurred in a patient who had a 15 mm diameter CBD stone in an 18 mm diameter CBD and was treated with precutting, a 10 mm diameter EPBD, and an ML. In a systematic review, EPLBD with EST is reported to carry a low risk of pancreatitis compared with EST or EPBD alone (2.4%, 4.3%, and 8.6%, respectively; *p* < 0.001) [46], therefore, a sufficient EPLBD for a dilated CBD may be important to avoid a risk of procedure-related pancreatitis, but further prospective studies will be needed, as described above.

In addition, patients enrolled in this study were relatively elderly, showing a median age of 79 years old. A previous study also reported that both technical success rates and the rates of adverse events were similar between elderly (≥75 years old) and non-elderly groups (<75 years old), suggesting that BE-ERCP is a feasible procedure for elderly individuals with a surgically altered anatomy [44]. Repeated BE-ERCP may carry a risk for elderly patients, so middle-term stent placement may be an option for the treatment of cases of complicated CBD stones, although caution against life-threatening cholangitis should be practiced [4,47].

Several limitations associated with the present study warrant mention. First, this was a retrospective study with a relatively small cohort, but three tertiary hospitals participated in it. Further prospective studies are needed to validate the present findings. Second, most of the patients were unable to be followed at the hospital, instead visiting family doctors. Therefore, an analysis based on long-term follow-up data, such as the stone recurrence rate, was not conducted.

In conclusion, therapeutic BE-ERCP for CBD stones in RYG patients, with a relatively elderly cohort, was effective and safe using short-type DBE. The largest CBD diameter ≥ 14 mm was an independent risk factor for failed complete stone extraction, but the use of a retroflex position may be considered as a recommended technique to achieve complete stone clearance.

## Figures and Tables

**Table 1:** Patients’ characteristics.

Patients/sessions, *n*	79/90
Age, years, median (IQR)	79 (73–84)
Sex, male/female, *n*	62/17
ASA-PS, 2/3/4, *n*	57/19/3
Reasons for gastrectomy, *n* (%)	
Gastric cancer	72 (91)
Esophageal cancer	1 (1)
Malignant lymphoma	1 (1)
Gastric ulcer	2 (3)
Unknown	3 (4)
Diameter of the largest CBD, mm, median (IQR)	14 (11–16)
Size of the largest CBD stone, mm, median (IQR)	10 (6–14)
Number of CBD stones, *n* (%)	
Debris	2 (3)
1	40 (51)
2	15 (19)
≥3	22 (28)

IQR, interquartile range; ASA-PS, American Society of Anesthesiologists physical status; CBD, common bile duct.

**Table 2:** Results of initial BE-ERCP (*n* = 79).

Reaching the papilla of Vatar, *n* (%)		73 (92)
Successful biliary cannulation, *n* (%)		64 (81)
Detection of stones by cholangiogram, *n* (%)		63 (80)
Overall procedure success, *n* (%)		62 (78)
Complete stone extraction, *n* (%)		42 (53)
Biliary stenting, *n* (%)		20 (25)
Time to reaching the papilla, min, median (IQR)		25 (11–40)
Time to biliary cannulation, min, median (IQR)		25 (6–33)
Total procedural time, min, median (IQR)		90 (67–120)
Adverse events, *n* (%)		4 (5)
Perforation	moderate/severe	1/1 (3)
Pancreatitis	mild	1 (1)
Hypoxia	mild	1 (1)

BE-ERCP, balloon enteroscopy-assisted endoscopic retrograde cholangiopancreatography; IQR, interquartile range.

**Table 3:** Details of ampullary procedure and biliary intervention in initial BE-ERCP (*n* = 63).

**Ampullary Procedure**	***n* (%)**
Precut alone	4 (6)
EST alone	6 (10)
EPBD alone	9 (14)
EPLBD alone	5 (8)
Precut + EPBD	12 (19)
Precut + EPLBD	5 (8)
EST + EPBD	12 (19)
EST + EPLBD	5 (8)
None	5 (8)
**Biliary Intervention**	***n*** **(%)**
Balloon catheter	46 (73)
Basket catheter	21 (29)
ML	22 (30)
Plastic stent	20 (27)
ENBD	2 (3)

EST, endoscopic sphincterotomy; EPBD, endoscopic papillary balloon dilation; EPLBD, endoscopic papillary large balloon dilation; ML, mechanical lithotripsy; ENBD, endoscopic nasobiliary drainage.

**Table 4:** Potential factors affecting complete stone extraction (*n* = 66, overall).

Variable	Complete Stone Extraction		Univariate		Multivariates	
	*n*	%	OR (95% CI)	*p* Value	OR (95% CI)	*p* Value
Age > 78 years old	28/34	82	1.56 (0.47–5.12)	0.55		
Male	46/57	81	2.09 (0.45–9.67)	0.39		
ASA-PS 3 or 4	13/16	81	1.22 (0.30–5.07)	>0.99		
Initial BE-ERCP	40/50	80	1.33 (0.35–5.03)	0.73		
Largest CBD diameter ≥ 14 mm	23/36	64	0.06 (0.01–0.50)	0.002	0.04 (0.003–0.58)	0.018
Retroflex position	26/29	90	3.67 (0.92–14.69)	0.073	6.43 (1.12–36.81)	0.037
Largest CBD stone size ≥ 10 mm	27/39	69	0.18 (0.04–0.89)	0.031	0.94 (0.11–8.15)	0.96
Number of CBD stones ≥ 3	13/19	68	0.44 (0.13–1.52)	0.20		
EST/Precut	36/43	84	2.25 (0.68–7.48)	0.21		
EPBD/EPLBD	46/59	78	0.59 (0.07–5.35)	>0.99		
Time from RYG to BE-ERCP > 4.9 years	26/33	79	1.00 (0.31–3.26)	>0.99		

ASA-PS, the American Society of Anesthesiologists physical status classification system; BE-ERCP, balloon enteroscope assisted-endoscopic retrograde cholangiopancreatography; CBD, common bile duct; EST, endoscopic sphincterotomy; EPBD, endoscopic papillary balloon dilation; EPLBD, endoscopic papillary large balloon dilation; RYG, Roux-en-Y gastrectomy; OR, odds ratio; CI, confidence interval.

**Table 5:** Potential factors associated with complete stone extraction (*n* = 36, Largest CBD diameter ≥14 mm).

Variable	Complete Stone Extraction		Univariate	
	*n*	%	OR (95% CI)	*p* Value
Age > 78 years old	9/15	60	0.75 (0.19–2.97)	0.74
Male	21/31	68	3.15 (0.45–21.95)	0.33
ASA-PS 3 or 4	4/7	57	0.70 (0.13–3.77)	0.69
Initial BE-ERCP	18/27	67	1.60 (0.34–7.46)	0.69
Retroflex position	15/18	83	6.25 (1.33–29.43)	0.035
Largest CBD stone size ≥ 10 mm	16/28	57	0.24 (0.03–2.22)	0.21
Number of CBD stones ≥ 3	10/16	63	0.90 (0.23–3.52)	>0.99
EST/Precut	16/22	73	2.67 (0.65–10.88)	0.29
EPBD/EPLBD	20/32	63	0.56 (0.05–5.97)	>0.99
Time from RYG to BE-ERCP > 4.9 years	14/21	67	1.33 (0.34–5.27)	0.74

ASA-PS, the American Society of Anesthesiologists physical status classification system; BE-ERCP, balloon enteroscope assisted-endoscopic retrograde cholangiopancreatography; CBD, common bile duct; EST, endoscopic sphincterotomy; EPBD, endoscopic papillary balloon dilation; EPLBD, endoscopic papillary large balloon dilation; RYG, Roux-en-Y gastrectomy; OR, odds ratio; CI, confidence interval.

**Table 6:** Potential factors associated with complete stone extraction (*n* = 39, largest CBD stone size ≥10 mm).

Variable	Complete Stone Extraction		Univariate	
	*n*	%	OR (95% CI)	*p* Value
Age > 78 years old	14/20	70	1.08 (0.28–4.20)	>0.99
Male	24/33	73	2.67 (0.45–15.72)	0.35
ASA-PS 3 or 4	8/11	73	1.26 (0.27–5.93)	>0.99
Initial BE-ERCP	20/28	71	1.43 (0.33–6.26)	0.71
Largest CBD diameter ≥ 14 mm	16/28	57	N.A.	0.017
Retroflex position	15/17	88	6.25 (1.15–34.12)	0.037
Number of CBD stones ≥ 3	9/15	60	0.50 (0.13–2.00)	0.48
EST/Precut	19/25	76	2.38 (0.59–9.64)	0.29
EPBD/EPLBD	25/36	69	1.14 (0.09–13.89)	>0.99
Time from RYG to BE-ERCP > 4.9 years	14/21	67	0.89 (0.23–3.54)	0.74

ASA-PS, the American Society of Anesthesiologists physical status classification system; BE-ERCP, balloon enteroscope assisted-endoscopic retrograde cholangiopancreatography; CBD, common bile duct; EST, endoscopic sphincterotomy; EPBD, endoscopic papillary balloon dilation; EPLBD, endoscopic papillary large balloon dilation; RYG, Roux-en-Y gastrectomy; OR, odds ratio; CI, confidence interval; N.A., not applicable.

## Data Availability

Data sharing is not applicable.
